# Multiple Pathogens Including Potential New Species in Tick Vectors in Côte d’Ivoire

**DOI:** 10.1371/journal.pntd.0004367

**Published:** 2016-01-15

**Authors:** Cyrille Bilé Ehounoud, Kouassi Patrick Yao, Mustapha Dahmani, Yaba Louise Achi, Nadia Amanzougaghene, Adèle Kacou N’Douba, Jean David N’Guessan, Didier Raoult, Florence Fenollar, Oleg Mediannikov

**Affiliations:** 1 Aix-Marseille Université, URMITE, UM63, CNRS 7278, IRD 198, Inserm U1095, Faculté de médecine, Marseille cedex 05, France; 2 Campus International UCAD-IRD, Dakar, Senegal; 3 Felix Houphouet Boigny Université, UFR Biosciences, Côte D’Ivoire; 4 Ecole de spécialisation en Elevage de Bingerville, Côte D’Ivoire; 5 Felix Houphouet Boigny Université, UFR Sciences médicales, Côte D’Ivoire; University of California, San Diego School of Medicine, UNITED STATES

## Abstract

**Background:**

Our study aimed to assess the presence of different pathogens in ticks collected in two regions in Côte d’Ivoire.

**Methodology/Principal Findings:**

Real-time PCR and standard PCR assays coupled to sequencing were used. Three hundred and seventy eight (378) ticks (170 *Amblyomma variegatum*, 161 *Rhipicepalus microplus*, 3 *Rhipicephalus senegalensis*, 27 *Hyalomma truncatum*, 16 *Hyalomma marginatum rufipes*, and 1 *Hyalomma impressum*) were identified and analyzed. We identified as pathogenic bacteria, *Rickettsia africae* in *Am*. *variegatum* (90%), *Rh*. *microplus* (10%) and *Hyalomma* spp. (9%), *Rickettsia aeschlimannii* in *Hyalomma* spp. (23%), *Rickettsia massiliae* in *Rh*. *senegalensis* (33%) as well as *Coxiella burnetii* in 0.2%, *Borrelia* sp. in 0.2%, *Anaplasma centrale* in 0.2%, *Anaplasma marginale* in 0.5%, and *Ehrlichia ruminantium* in 0.5% of all ticks. Potential new species of *Borrelia*, *Anaplasma*, and *Wolbachia* were detected. *Candidatus* Borrelia africana and *Candidatus* Borrelia ivorensis (detected in three ticks) are phylogenetically distant from both the relapsing fever group and Lyme disease group borreliae; both were detected in *Am*. *variegatum*. Four new genotypes of bacteria from the *Anaplasmataceae* family were identified, namely *Candidatus* Anaplasma ivorensis (detected in three ticks), *Candidatus* Ehrlichia urmitei (in nine ticks), *Candidatus* Ehrlichia rustica (in four ticks), and *Candidatus* Wolbachia ivorensis (in one tick).

**Conclusions/Significance:**

For the first time, we demonstrate the presence of different pathogens such as *R*. *aeschlimannii*, *C*. *burnetii*, *Borrelia* sp., *A*. *centrale*, *A*. *marginale*, and *E*. *ruminantium* in ticks in Côte d’Ivoire as well as potential new species of unknown pathogenicity.

## Author Summary

The management of febrile illnesses represents a veritable challenge in sub Saharan-Africa. Until recently most of them were considered as malaria. However, it was showed that a large part of non-malarial febrile diseases in African rural regions (for instance, in Senegal) may be caused by tick-borne infections. Unfortunately, no data exist about the prevalence and incidence of tick-borne diseases in Côte d'Ivoire and their role in public health. We aimed to search for different pathogenic bacteria in ticks in order to understand if there is the background for tick-borne diseases. We detected pathogenic bacteria responsible for many infectious diseases like *Rickettsia* (spotted fevers), *Borrelia* (relapsing fevers), *Anaplasma*, *Ehrlichia* (ehrlichiosis and anaplasmosis) and *Coxiella burnetii* (Q fever). These finding suggested that, as in others sub-Saharan African countries, tick-borne disease may be considered as a health care problem in Cote d'Ivoire.

## Introduction

Ticks are important vectors of many pathogens and are considered as the second biggest vectors of human and animal diseases after mosquitoes [1,2]. Many tick-borne bacterial emerging diseases such as spotted fevers, borrelioses, anaplasmoses, ehrlichioses, and Q fever have been described worldwide [3,4,5]. It was recently shown that in many tropical countries tick- and acari-borne infections play important role in human pathology. In Senegal, for instance, arthropod-borne borreliosis and rickettsiosis were identified in 16.3% of acute fevers recorded by rural dispensaries [6]. Acari-borne tsutsugamushi fever is one of the major causes of acute febrile morbidity in South-Eastern Asia [7]. Investigations of the vectors of tick-borne diseases are one of the main keys to controlling related morbidity [8].

Rickettsioses, caused by bacteria belonging to the spotted fever group (SFG) of the genus *Rickettsia*, are considered among the oldest known vector-borne zoonotic diseases [9]. The most common rickettsia in Africa is *Rickettsia africae*, the etiological agent of African tick-borne fever [10]. This disease has been reported with high seroprevalence in sub-Saharan African countries including Cameroon (11.9% - 51.8%) and Senegal (21.4% - 51%) [11,12]. *R*. *africae* has been detected by PCR in ticks in Mali, Niger, Burundi, and Sudan [13]. *Amblyomma hebraeum* and *Amblyomma variegatum* ticks are the main reservoirs and vectors of *R*. *africae* in Southeastern Africa and sub-Saharan Africa, respectively [9,14]. It was also reported in other species of *Amblyomma* such as *Amblyomma lepidum* in Djibouti [15] and *Amblyomma compressum* in the Democratic Republic of Congo and Liberia [16,17]. In Western Africa, *R*. *africae* has been detected in several *Rhipicephalus* ticks including *Rhipicephalus annulatus* in Guinea, Senegal, and Nigeria [12,16,18], *Rhipicephalus evertsi evertsi* in Senegal and Nigeria [12,18], *Rhipicephalus decoloratus* in Nigeria [19], *Rhipicephalus geigyi* in Liberia [16], and *Hyalomma* spp. ticks including *Hyalomma impeltatum* in Nigeria [18] and *Hyalomma marginatum rufipes* in Guinea [16] but not in Côte d’Ivoire, where a strain of *R*. *africae* has been isolated from *Am*. *variegatum* [20].

*Rickettsia aeschlimannii* is an agent of spotted fever which was first identified in a patient returning from Morocco [21]. In this country, it was first isolated from *Hyalomma marginatum marginatum* ticks [22]. *R*. *aeschlimannii* was also reported by PCR in other *Hyalomma* ticks including *H*. *marginatum rufipes* and *Hyalomma truncatum* ticks collected from camels and cows in Egypt, Algeria, Sudan, and Tunisia [23]. In Western Africa, *R*. *aeschlimannii* was also detected in 15% to 95% of *H*. *marginatum rufipes* from Mali, Niger, Senegal and Nigeria [12,13,24] and in 6% to 7% of *H*. *truncatum* from Senegal [12] but not in Côte d’Ivoire. *Rickettsia massiliae* is another SFG rickettsia. Since its description in 2005, *R*. *massiliae* infections in humans have been confirmed in Europe and South America [25,26,27]. It is associated with *Rhipicephalus* ticks. *R*. *massiliae* was found by PCR in *Rhipicephalus* spp. ticks including *Rhipicephalus* spp. from Côte d’Ivoire [28], *Rhipicephalus guilhoni* from Senegal [12], *Rhipicephalus senegalensis* from Guinea [16], and *Rhipicephalus eversti* from Nigeria [18].

Different borrelioses are caused by bacteria from the *Borrelia* genus. They are traditionally classified into the Lyme disease group and the relapsing fever group. The former is ecologically associated with hard ticks and is mostly found in the temperate northern hemisphere [29]. Relapsing fever group borreliae are mostly associated with soft ticks and found in subtropical regions worldwide [30,31]. In endemic regions, borrelioses may play an important role, for example in Slovakia [32]. Relapsing fever is one of the most common diseases in several African regions including Senegal [33,34] and east African countries [35]. It is caused by different *Borrelia* species such as *Borrelia hispanica*, *Borrelia duttonii*, and *Borrelia crocidurae*. *B*. *hispanica* was recently detected in 11.6% to 20% of *Ornithodoros* ticks from northern Africa [31,36]. *B*. *crocidurae* is responsible for tick-borne relapsing fever in West Africa. Its distribution in the south is thought to be limited by the 750 mm isohyets [37]. Neither this borrelia nor any other from the relapsing group has been reported in Côte d’Ivoire. A controversial study, based on molecular data, reported 30 cases of borreliosis in Togo but its epidemiology was not identified [38] and studies in neighboring countries did not confirm the presence of borreliosis in west tropical sub-Saharan Africa. In Ethiopia, *Borrelia* sp. was recently identified by PCR in 7.3% of *Amblyomma cohaerens* [39]. Phylogenetically, this *Borrelia* sp. was placed in an intermediate position between Lyme disease and relapsing fever groups.

All bacteria from the *Anaplasmataceae* family are intracellular mammal parasites, arthropods nematodes, and trematodes [40]. *Anaplasma centrale* and *Anaplasma marginale* are two etiological agents of bovine anaplasmosis in ruminants [41]. These species are distributed in tropical and subtropical regions of Africa and naturally infect cattle [42]. They were previously found by molecular biology in ticks in neighboring Mali [43]. These bacteria are often found in *Dermacentor*, *Rhipicephalus*, and *Amblyomma* ticks throughout the world [44]. *Ehrlichia ruminantium* is responsible for cowdriosis in ruminants with the *Amblyomma* genus ticks as a vector [45]. Cowdriosis induces mortality in ruminants in sub-Saharan Africa and in islands in the Caribbean where it causes serious losses to animal production [40]. *E*. *ruminantium* was previously identified in *Am*. *variegatum* in Burkina Faso but not in Côte d’Ivoire. No cases of human ehrlichiosis or anaplasmosis have been reported in Africa, but recently human pathogens such as *Anaplasma phagocytophilum* have been reported in Senegal and Algeria [46,47]. Bacteria from the *Wolbachia* genus of the *Anaplasmatacae* family are associated with arthropods and filarial nematodes. They are responsible for reproductive alterations in arthropods which are indirectly (via nematodes) associated with human pathogenesis [40].

Finally, Q fever is a zoonotic disease caused by *Coxiella burnetii*. This bacterium may cause severe infections such as chronic endocarditis and abortion [48,49]. It infects humans usually by a direct contact with domestic animals such as cattle, sheep, goats, and dogs [50]. It was previously reported in *Amblyomma*, *Rhipicephalus*, and *Dermacentor* ticks [43]. In Senegal, *C*. *burnetii* was detected in 0.8% to 14.2% of ticks including *Am*. *variegatum*, *Rhipicephalus* spp., *Hyalomma* spp., and *Ornithodoros sonrai* [51] and may play a role in Q fever epidemiology. In Côte d’Ivoire, the seroprevalence was estimated at 3.4% [52].

Although these diseases have emerged in many African countries, they remain neglected. In Côte d’Ivoire, little information is available about these diseases and their epidemiology. To date, the existence and/or prevalence of tick-borne associated pathogens remain poorly understood. Our study provides the first data screening for multiple tick-borne associated pathogens in Côte d’Ivoire.

## Materials and Methods

### Ethics statement

To perform this study, an approval of Cote d'Ivoire Ethics committee was received under the number N°86/MSLS/CNERN-dkn.

### Period, study area and tick collection

**Fig 1:**
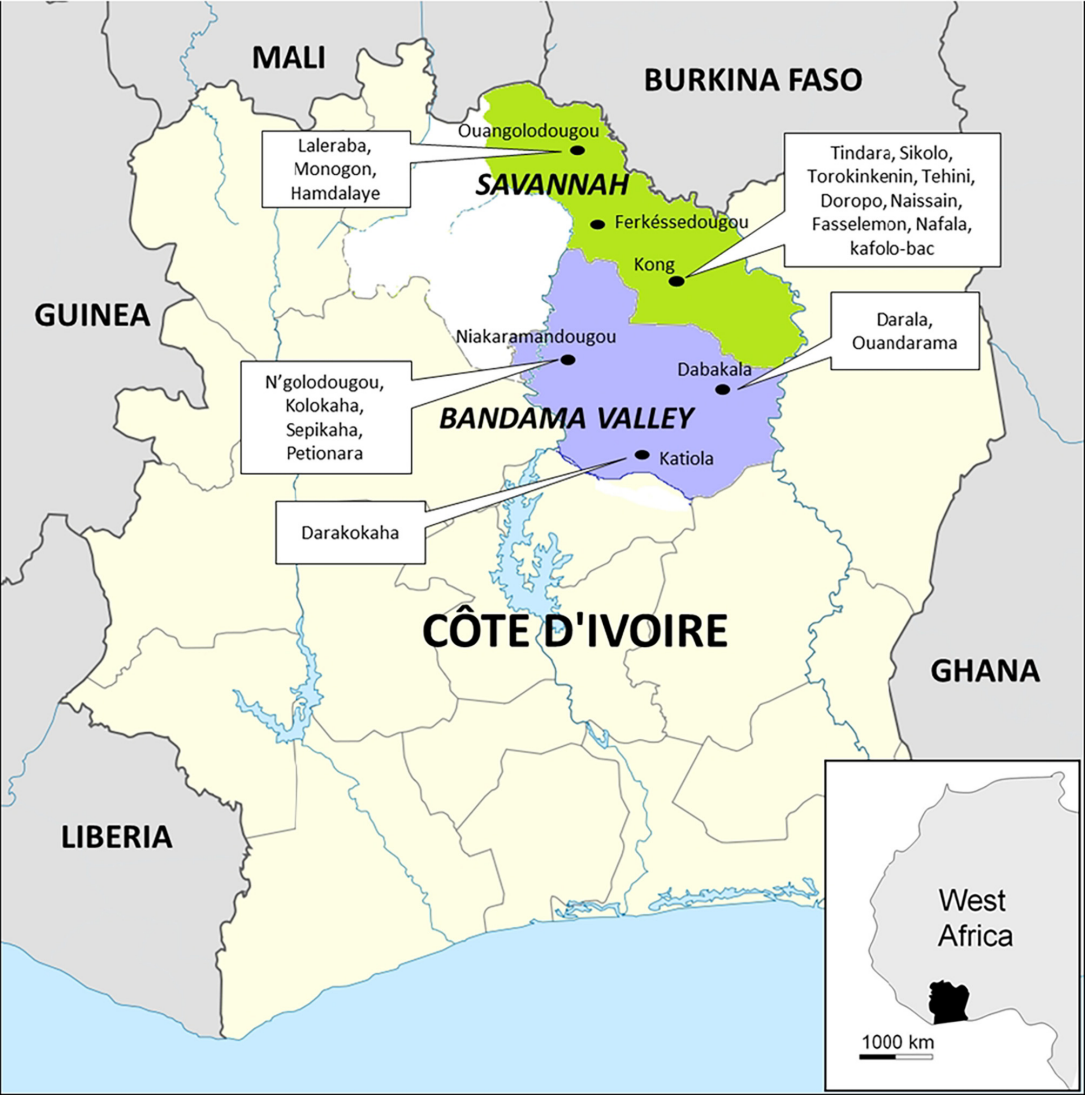
Map of Côte d’Ivoire showing the regions, cities and villages where the ticks were collected for our study.

The tick collection was conducted over a period ranging from October 30 to November 8, 2014. Ticks were manually collected from cattle in two regions of Côte d'Ivoire: Savannah and Bandama Valley (Fig 1, Table 1). In total, 378 ticks (304 adults and 74 nymphs) were collected from three cities and 12 villages in the Savannah region and three cities and seven villages in the Bandama Valley region (Table 1). Ticks were stored in 70% ethanol until morphological and molecular analyses in laboratory of URMITE, Marseille (France). The species and sex of the ticks were identified according to standard taxonomic keys for adult ticks [2].

**Table 1:** Geographic coordinates of tick collection sites.

City^1^ or village^2^	Geographic coordinates	Species	Number (male/female/nymphs)	Total number
**Savannah region**				
Kong^**1**^	08°15N 05°07W	*Rh*. *microplus*	3/10/0	13
Ferké^**1**^	09°55N 05°20W	*Am*. *variegatum*	7/3/4	14
		*Rh*. *microplus*	1/4/0	5
		*H*. *impressum*	1/0/0	1
		*H*. *truncatum*	0/1/0	1
Kafolo-bac^2^	09°43N 04°39W	*Am*. *variegatum*	1/0/5	6
		*Rh*. *microplus*	3/8/1	12
		*H*. *truncatum*	1/0/0	1
Torokinkenin^2^	08°84N 04°47W	*Am*. *variegatum*	2/2/1	5
		*Rh*. *microplus*	4/10/0	14
Téhini^2^	09°60N 03°67W	*Am*. *variegatum*	2/1/0	3
		*Rh*. *microplus*	1/4/1	6
		*H*. *marginatum rufipes*	0/1/0	1
Doropo^2^	09°77N 03°40W	*Am*. *variegatum*	2/0/9	11
		*Rh*. *microplus*	0/7/0	7
		*H*. *marginatum rufipes*	2/2/0	4
		*H*. *truncatum*	0/2/0	2
Naissain^2^	09°39N 04°48W	*Am*. *variegatum*	0 /1/4	5
		*Rh*. *microplus*	1/4/0	5
Sikolo^2^	09°43N 04°66W	*Am*. *variegatum*	0/0/4	4
		*Rh*. *microplus*	1/4/0	*5*
Fasselemon^2^	09°27N 04°52W	*Am*. *variegatum*	4/1/0	5
		*Rh*. *microplus*	1/4/0	5
Nafana^2^	09°18N 04°78W	*Am*. *variegatum*	1/0/0	1
		*Rh*. *microplus*	1/1/0	2
Tindara^2^	09°54N 04°74W	*Am*. *variegatum*	4/2/11	17
		*Rh*. *microplus*	0/12/0	12
		*H*. *truncatum*	1/0/0	1
Laleraba^2^	10°13N 05°08W	*Am*. *variegatum*	4/1/0	5
		*Rh*. *microplus*	0/5/0	5
		*H*. *truncatum*	7/3/0	10
		*H*. *marginatum rufipes*	1/2/0	3
Hamdalaye^2^	09°98N 05°13W	*Am*. *variegatum*	10/6/3	19
		*Rh*. *microplus*	1/4/0	5
		*H*. *truncatum*	7/2/0	9
		*H*. *marginatum rufipes*	2/1/0	3
Monogon^2^	09°82N 04°92 W	*Am*. *variegatum*	2/3/0	5
		*Rh*. *microplus*	2/4/0	6
		*H*. *truncatum*	1/2/0	3
		*H*. *marginatum rufipes*	2/3/0	5
**Bandama Valley region**				
Dabakala^1^	08°36N 04°41W	*Am*. *variegatum*	10/2/2	14
		*Rh*. *microplus*	2/4/0	6
Katiola^1^	08°15N 05°07W	*Am*. *variegatum*	3/0/4	7
		*Rh*. *microplus*	1/4/0	5
Niakara^1^	06°60N 05°29W	*Am*. *variegatum*	8/2/9	19
		*Rh*. *microplus*	3/2/0	5
Darala^2^	08°44N 04°35W	*Am*. *variegatum*	2/1/0	3
		*Rh*. *microplus*	1/4/0	5
		*Rh*. *senegalensis*	1/1/0	2
Ouandarama^2^	08°68N 04°39W	*Am*. *variegatum*	2/0/4	6
		*Rh*. *microplus*	1/4/1	6
		*Rh*. *senegalensis*	0/1/0	1
Darakokaha^2^	08°27N 05°16W	*Am*. *variegatum*	1/0/0	1
		*Rh*. *microplus*	1/4/0	5
N’golodougou^2^	09°15N 05°12W	*Am*. *variegatum*	4/2/0	6
		*Rh*. *microplus*	2/7/0	9
Kolokaha^2^	08°97N 05°21W	*Am*. *variegatum*	2/2/0	4
		*Rh*. *microplus*	1/4/0	5
Sépikaha^2^	08°91N 05°03W	*Am*. *variegatum*	1/0/9	10
		*Rh*. *microplus*	2/4/2	8
Petionara^2^	08°47N 05°03W	*Rh*. *microplus*	1/4/0	5
		*Am*. *variegatum*	72/29/69	170
		*Rh*. *microplus*	34/122/5	161
		*Rh*. *senegalensis*	1/2/0	3
		*H*. *truncatum*	17/10/0	27
		*H*. *marginatum rufipes*	7/9/0	16
		*H*. *impressum*	1/0/0	1
**Total of ticks for the two regions**			**132/172/74**	**378**

### DNA extraction and real-time PCR

Total DNA from half of each tick was extracted using the EZ1 DNA tissue kit (Qiagen, Hilden, Germany) following the manufacturer’s instructions. DNA extracts were stored at +4°C until use. Bacterial DNA was initially detected using bacterial genus-specific or species-specific quantitative real-time PCRs (qPCRs) targeting: *Rickettsia* spp., *R*. *africae*, *R*. *aeschlimannii*, *R*. *massiliae*, *Borrelia* spp., *Anaplasmataceae* spp., *A*. *phagocytophilum*, *Bartonella* spp., *C*. *burnetii*, and *Spiroplasma* spp. (Table 2). Samples with a high discordance in the cycle threshold number (Ct) for *Rickettsia* spp. and *R*. *africae* (in all cases, low Ct for *Rickettsia* spp. and high Ct for *R*. *africae*) were subjected to specific qPCRs for two other rickettsial species: *R*. *aeschlimannii* and *R*. *massiliae* in order to identify possible co-infection. qPCRs were performed using a CFX 96 Real Time System (Bio-Rad, Marnes-la-Coquette, France) and the Eurogentec MasterMix Probe PCR kit (Eurogentec, Liège, Belgium). PCR tests were considered to be positive when the Ct was lower than 35 Ct [22]. In addition, two different specific qPCRs targeting two different sequences had to be positive in order to confirm the presence of a bacterium in the ticks. Positive controls (bacterial DNA) and negative controls (master mix or water) were used to validate the PCR runs.

**Table 2:** Primers and probes used for real-time quantitative PCR in this study.

Microorganisms	Targeted sequence	Primers f, r (5’-3’) and Probes p (6FAM–TAMRA)	References
*Rickettsia* spp.	*gltA* (RKNDO3)	f_GTGAATGAAAGATTACACTATTTAT	[53]
		r_GTATCTTAGCAATCATTCTAATAGC	
		p_CTATTATGCTTGCGGCTGTCGGTTC	
*R*. *africae*	poT15-dam2	f_TGCAACACGAAGCACAAAAC	[6]
		r_CCTCTTGCGAAACTCTACTT	
		p_TGA CGTGTGGATTCGAGCACCGGA	
*R*. *aeschlimannii*	Intergenic spacer (RaescSca1)	f_AAAGAAATGGATTTCACGGCGAA	[12]
		r_ACCAAGTAAACGTCTCGTAC	
		p_TGGGGAAATATGCCGTATACGCAAGC	
*R*. *massiliae*	Hypothetical protein	f_CCAACCTTTTGTTGTTGCAC	[54]
		r_TTGGATCAGTGTGACGGACT	
		p_CACGTGCTGCTTATACCAGCAAACA	
*Anaplasma* spp.	23S rRNA (TtAna)	f_TGACAGCGTACCTTTTGCAT	[47]
		r_TGGAGGACCGAACCTGTTAC	
		p_GGATTAGACCCGAAACCAAG	
*Anaplasma phagocytophilum*	apaG	f_TAAGCGCAGTTGGAAGATCA	[55]
		r_CGGCACATCCACATAAAACA	
		p_TGATGAACGGCTGGTATCAG	
*Spiroplasma*	*rpoB*	f_TGTTGGACCAAACGAAGTTG	[55]
		r_CCAACAATTGGTGTTTGTGG	
		p_GCTAACCGTGCTTTAATGGG	
*Coxiella burnetii*	Insertion Sequence (IS1111)	f_CAAGAAACGTATCGCTGTGGC	[56]
		r_CACAGAGCCACCGTATGAATC	
		p_CCGAGTTCGAAACAATGAGGGCTG	
	(IS30A)	f_CGCTGACCTACAGAAATATGTCC	[57]
		r_GGGGTAAGTAAATAATACCTTCTGG	
		p_CATGAAGCGATTTATCAATACGTGTATG	
*Bartonella* spp.	Internal transcribed spacer16S (BartoITS3)	f_GATGCCGGGGAAGGTTTTC	[58]
		r_GCCTGGGAGGACTTGAACCT	
		p_GCGCGCGCTTGATAAGCGTG	
*Borrelia* spp	Internal transcribed spacer 16S RNA (Bor ITS4)	f_GGCTTCGGGTCTACCACATCTA	[59]
		r_CCGGGAGGGGAGTGAAATAG	
		p_TGCAAAAGGCACGCCATCACC	
	(Bor_16S)	f_AGCCTTTAAAGCTTCGCTTGTAG	[34]
		r_GCCTCCCGTAGGAGTCTGG	
		p_CCGGCCTGAGAGGGTGAACGG	

### Standard PCR and sequencing

Most of samples which were considered positive by qPCRs were subsequently subjected to standard PCR. All samples which were positive using *Rickettsia* genus-specific but negative with *R*. *africae* qPCR were subjected to standard PCR to amplify a portion of the *ompA* gene. We also chose two positive ticks for *R*. *africae* by species to confirm the presence of *R*. *africae* by standard PCR. The primers used (190.70, 190.180, and 190.701) amplified a 632-bp fragment of the *Rickettsia ompA* gene [60]. For the identification of *Borrelia* species, primers targeting a portion of the *flaB* gene were used [33]. *Anaplasmataceae* spp. (*Anaplasma* spp., *Ehrlichia* spp., and *Wolbachia* spp.) were identified using Ana 212f and Ana 753r primers targeting a 500 bp portion of the 23S rRNA gene [47].

Standard PCR was performed on a ThermalCycler (Applied Biosystem, Paris, France). The reactions were carried out using the Hotstar Taq-polymerase (Qiagen), in accordance with the manufacturer’s instructions. The amplicons were visualized using electrophoresis on a 1.5% agarose gel stained with ethidium bromide and examined using an ultraviolet transilluminator. The PCR products were purified using a PCR filter plate Millipore NucleoFast 96 PCR kit following the manufacturer’s recommendations (Macherey–Nagel, Düren, Germany). The amplicons were sequenced using the BigDye Terminator Cycle Sequencing Kit (Applied Biosystems) with an ABI automated sequencer (Applied Biosystems).The sequences which were obtained were assembled using ChromasPro software (ChromasPro 1.7, Technelysium Pty Ltd.,Tewantin, Australia) and compared with those available in GenBank by NCBI BLAST (http://blast.ncbi.nlm.nih.gov/Blast.cgi).

### Phylogenetic analysis

DNA sequences alignment was carried out using MEGA 6 (http://www.megasoftware.net/mega.php). We selected the Bayesian method [61] using TOPALi 2.5 software (Biomathematics and Statistics Scotland) to construct phylogenetic trees.

## Results

Of the 378 ticks identified, 170 *Am*. *variegatum*, 161 *Rh*. *microplus*, 3 *Rh*. *senegalensis*, 27 *H*. *truncatum*, 16 *H*. *marginatum rufipes*, and one *H*. *impressum* were analyzed. No *A*. *phagocytophilum*, *Bartonella* spp. and *Spiroplasma* spp. were detected in ticks. *Rickettsia* spp. was found in 187 of 378 ticks (49%); most of them, 174/378 (46%), were identified as *R*. *africae* with specific qPCR (Table 3). *R*. *africae* was detected in 154/170 (90%) *Am*. *variegatum*, 16/161 (10%) *Rh*. *microplus*, 2/16 (12%) *H*. *marginatum rufipes*, 1/27 (4%) *H*. *truncatum* and 1/1 *H*. *impressum* (Table 3). To confirm the presence of *R*. *africae*, we performed standard PCR using two positive ticks per species. The BLAST search of the *ompA* gene sequences from ticks revealed 100% nucleotide identity with the *ompA* gene of *R*. *africae* detected in *Am*. *variegatum* collected in Antigua (GenBank EU622980). We amplified the *ompA* fragment in all ticks positive for *Rickettsia* spp. but negative for *R*. *africae* qPCR. The BLAST analyses showed that *ompA* sequences of *R*. *aeschlimannii* were detected in 7/16 (44%) *H*. *marginatum rupifes* and 3/27 (11%) *H*. *truncatum*. The sequences were 99% identical to those of *R*. *aeschlimannii*, previously detected in *H*. *impeltatum* collected in Egypt (GenBank HQ335157) and 100% identical to those detected in *H*. *marginatum* in Turkey (GenBank KF791251). *R*. *massiliae* was observed in 1/3 (33%) *Rh*. *senegalensis* with 100% similarity *R*. *massiliae*, previously detected in *Rh*. *senegalensis* in Guinea (GenBank JN043508). Finally, these results were confirmed by a specific qPCR for *R*. *aeschlimannii* and *R*. *massiliae* (Table 2). We also performed these species-specific qPCR on three samples (two *H*. *marginatum rufipes* and one *Rh*. *senegalensis*) where we observed a high discordance (more than 5 Cts) between *Rickettsia* genus-specific qPCR (low Ct) and *R*. *africae* species-specific qPCR (higher Ct). We found that in all three cases, a co-infection by two rickettsia species: *R*. *massiliae* plus *R*. *africae* in *Rh*. *senegalensis* and *R*. *aeschlimannii* plus *R*. *africae* in *H*. *marginatum rufipes*.

**Table 3:** Prevalence of positive ticks by PCR.

Bacterium%**(positives/tested)**	*Am*. *variegatum*	*Rh*. *microplus*	*Rh*. *senegalensis*	*H*. *truncatum*	*H*. *marginatum*	*H*. *impressum*	Total
*Rickettsia* spp.	90% (154/170)	10% (16/161)	33% (1/3)	15% (4/27)	69% (11/16)	100% (1/1)	49% (187/378)
*R*. *africae*	90% (154/170)	10% (16/161)	-	4% (1/27)	12% (2/16)	100% (1/1)	46% (174/378)
*R*. *aeschlimannii*	-	-	-	11% (3/27)	44% (7/16)	-	2% (10/378)
*R*. *massiliae*	-	-	33% (1/3)	-	-	-	0.2% (1/378)
*C*. *burnetii*	0.6% (1/170)	-	-	-	-	-	0.2% (1/378)
*Borrelia* spp.	6% (11/170)	2% (3/161)	-	4% (1/27)	6% (1/16)	-	5% (16/378)
*Borrelia* sp. genotype TCI301	-	0.6% (1/161)	-	-	-	-	0.2% (1/378)
*Candidatus* Borrelia ivorensis	1% (2/170)	-	-	-	-	-	0.5% (2 /378)
*Candidatus* Borrelia africana	0.6% (1/170)	-	-	-	-	-	0.2% (1/378)
*Anaplasma* spp.	12% (21 /170)	24% (39/161)	-	11% (3/27)	-	-	16% (63/378)
*Anaplasma centrale*	0.6% (1/170)	-	-	-	-	-	0.2% (1/378)
*Anaplasma marginale*	-	1% (2/161)	-	-	-	-	0.5% (2/378)
*Candidatus* Anaplasma ivorensis	2% (3/170)	-	-	-	-	-	0.8% (3/378)
*Ehrlichia* sp.	3% (6/170)	3% (5/161)	-	7% (2/27)	-	-	3% (13/378)
*Candidatus* Ehrlichia rustica	0.6% (1/170)	1% (2 /161)	-	4% (1/ 27)	-	-	1% (4/378)
*Candidatus* Ehrlichia urmitei	3% (5/170)	2% (3/161)	-	4% (1/27)	-	-	2% (9/378)
*Ehrlichia ruminantium*	1% (2/170)	-	-	-	-	-	0.5% (2/378)
*Candidatus* Wolbachia ivorensis	-	0.6% (1 /161)	-	-	-	-	0.2% (1/378)
*R*. *africae +R*.*aeschlimannii*	-	-	-	-	12% (2/16)	-	0.5% (2/378)
*R*. *africae +R*. *massiliae*	-	-	33% (1/3)	-	-	-	0.2% (1/378)
*R*. *africae +C*. *burnetii*	0.6% (1/170)	-	-	-	-	-	0.2% (1/378)
*R*. *africae +Borrelia* sp.	-	0.6% (1/161)	-	-	-	-	0.2% (1/378)
*R*. *africae + Candidatus* Borrelia Africana *+ Candidatus* Borrelia ivorensis	2% (3/170)	-	-	-	-	-	0.8% (3/378)
*R*. *africae +Anaplasma centrale*	0.6% (1/170)	-	-	-	-	-	0.2% (1/378)
*R*. *africae +Anaplasma marginale*	-	-	-	-	-	-	0.2% (1/378)
*R*. *africae +Candidatus* Anaplasma ivorensis	1% (2/170)	0.6% (1/161)	-	-	-	-	0.8% (3/378)
*R*. *africae + Candidatus* Ehrlichia urmitei	1% (2/170)	-	-	-	-	-	0.5% (2/378)

= 0%; the name *‘Candidatus’* is employed here for the new species because they are not isolated

**Fig 2:**
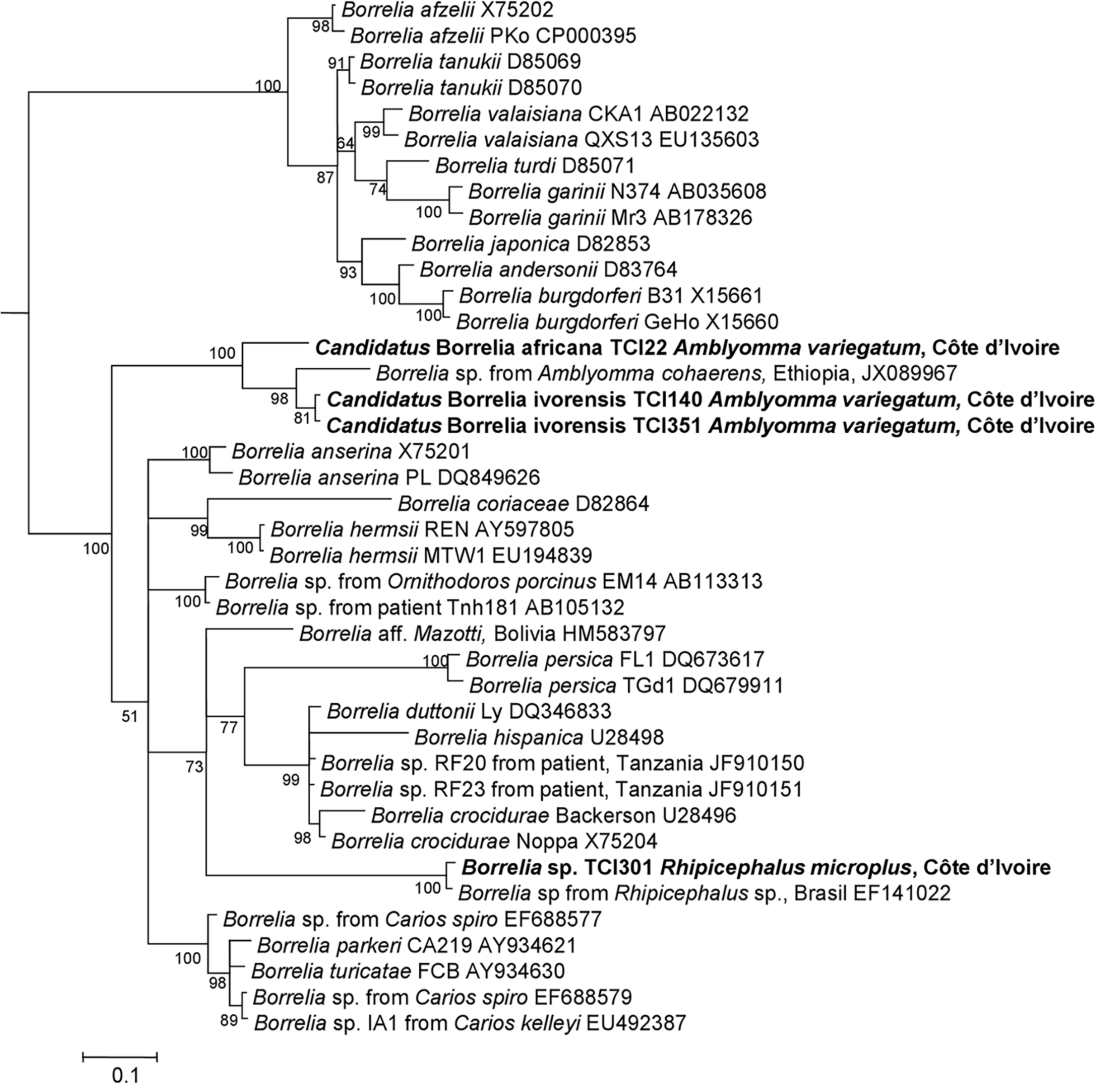
*flaB* gene-based phylogenetic analysis of the strains identified in this study. Phylogenetic tree highlighting the position of *Borrelia* sp. identified in the present study relative to *borrelia* type strains and uncultured borreliae. The *flaB* sequences were aligned using CLUSTALW, and phylogenetic inferences were obtained from a Bayesian phylogenetic analysis with the HKY+Γ; JC+ Γ and HKY+ Γ substitution models for the first, second and third codons respectively. The GenBank accession numbers are indicated at the end. Sequences obtained in the present study are in bold. The numbers at the nodes are the bootstrap values obtained by repeating the analysis 100 times to generate a majority consensus tree. There were a total of 300 positions in the final dataset. The scale bar indicates a 10% nucleotide sequence divergence.

*C*. *burnetii* was detected in one tick (Table 3). Screening of all ticks for *Borrelia* spp. using qPCR, detected 16/378 (4%) positive ticks. We succeeded in amplifying a fragment of *flaB* gene and 16S rRNA sequence only in 4/378 (1%) ticks. A BLAST search showed that these sequences probably belong to an undescribed species, because only 87% (288/329 bp), 87% (287/328 bp), 97% (319/328 bp), and 87% (288/329 bp) similarities were observed with, respectively, the *flaB* gene of *Borrelia duttonii* (GenBank AB105132), *Borrelia* sp. IA-1 (GenBank EU492387), *Borrelia* sp. BrFlab (GenBank EF141022), and *Borrelia* sp. IA-1 (GenBank EU492387). The phylogenetic position of this *Borrelia* is shown in Fig 2. Because these potentially new species had not previously been isolated, we propose the provisional names *Candidatus* Borrelia africana for the genotype TCI22 and *Candidatus* Borrelia ivorensis for the genotypes TCI140 and TCI351. In a phylogenetic tree based on a 344 bp fragment of the *Borreliae flaB* gene, the sequences of *Candidatus* Borrelia africana and *Candidatus* Borrelia ivorensis are situated in the *Borrelia* genus near *Borrelia* sp. from Ethiopian *Amblyomma cohaerens* (GenBank JX089967) and are closer to the relapsing fever group than to that of Lyme disease. As previously shown, Ethiopian *Borrelia* group together with these new genotypes to form a separate and well-supported (bootraps 100) branch on the phylogenetic tree situated between Lyme disease and relapsing fever clusters, albeit closer to the latter. We also identified *Borrelia* sp. (genotype TCI301) in *Rh*. *microplus* which was almost identical to *Borrelia* sp. previously identified in the same ticks in Brazil (GenBank EF141022).

**Fig 3:**
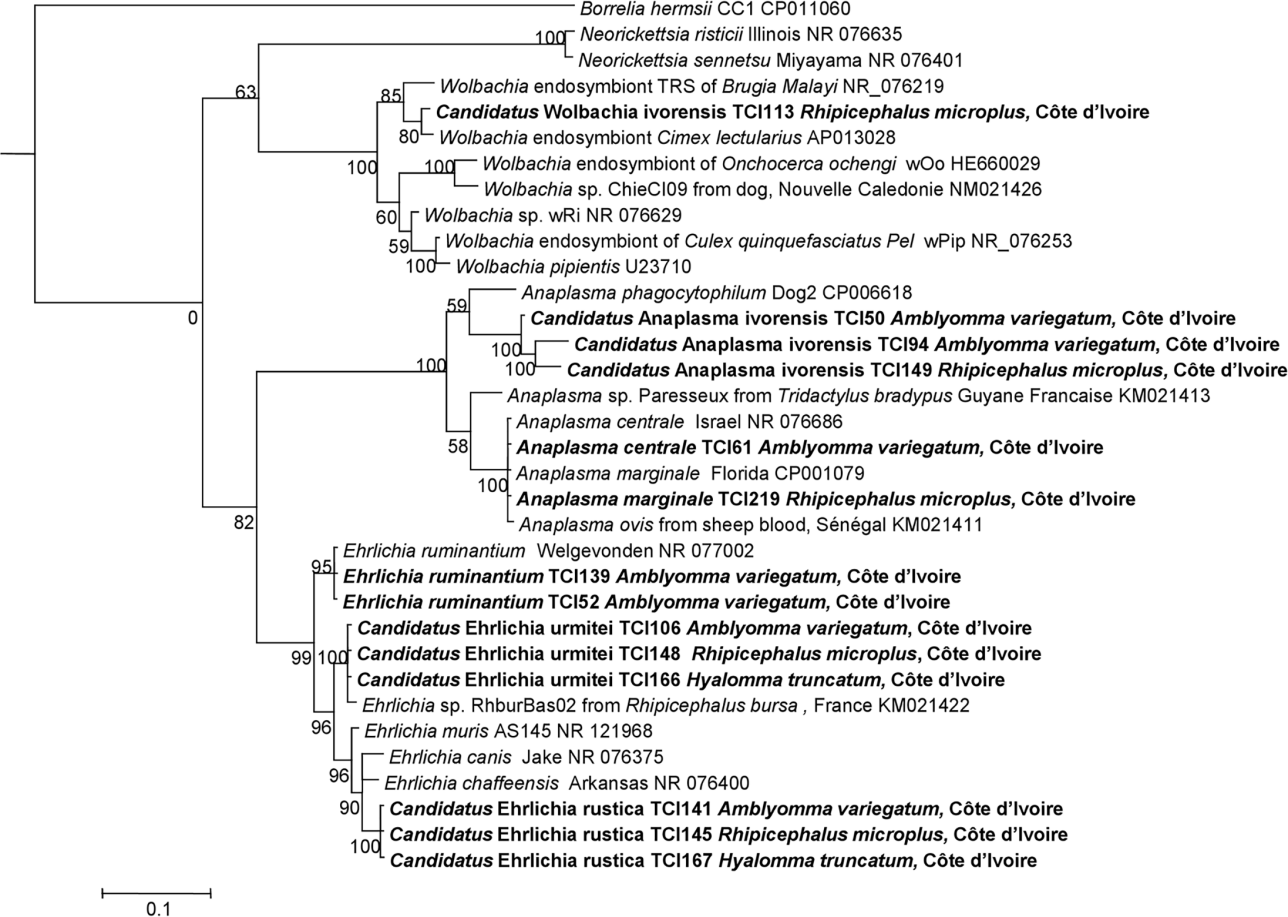
23S rRNA based phylogenetic analysis of strains identified in this study. Phylogenetic tree highlighting the position of *Anaplasma* sp, *Ehrlichia* sp and *Wolbachia* sp identified in the present study relative to *Anaplama*, *Ehrlichia* and *Wolbachia* type and uncultured strains. The 23S rRNA sequences were aligned using MEGA 6 and phylogenetic inferences were obtained from a Bayesian phylogenetic analysis with the HKY standard model.

Sixty-three ticks were positive using qPCR targeting the 23S rRNA of *Anaplasmataceae*. Only 39 DNA samples were positive using qPCR were successfully amplified in standard PCR. A possible explanation may consist of the lower sensitivity of standard PCR compared to qPCR. After sequencing, we obtained good quality sequences for only 22 samples (22/378; 6%). We suggest that the poor sequence quality may be explained by co-infection by two or more species belonging to the *Anaplasmataceae* family. We have identified one case of *A*. *centrale* in *Am*. *variegatum* (100% identity with the *A*. *centrale* strain Israel, NR_076686), and two cases of *E*. *ruminantium* in *Am*. *variegatum* (100% identity with the *E*. *ruminantium* strain Welgevonden, NR_077000). We have identified *A*. *marginale* in two *Rh*. *microplus* (100% of homology with *A*. *marginale* strain Florida, NR_0765879). Finally, for all remaining sequences, Blast analysis shows a homology score of under 92% which means that these sequences are likely to correspond to new species. After the construction of a phylogenetic tree (Fig 3), we propose that the status of *Candidatus* is applied to an uncultured species but not formally recognized by the International Code of Nomenclature of Bacteria [62]. The result shows three cases of *Anaplasma*: *Candidatus* Anaplasma ivorensis related to *A*. *phagocytophilum* identified in ticks, two in *Am*. *variegatum*, and one in *Rh*. *microplus*. The three sequences have one to two SNP (single nucleotide polymorphism) between them. In one *Rh*. *microplus*, a potential new *Wolbachia* sp., *Candidatus* Wolbachia ivorensis, was identified, closely related to the *Wolbachia* endosymbiont of *Cimex lectularius* (GenBank AP013028). We also identified two groups of sequences corresponding to new *Ehrlichia* spp. which cluster in two clades. Indeed, in four cases (one *Am*. *variegatum*, two *Rh*.*microplus*, and one *H*. *truncatum*), we identified *Candidatus* Ehrlichia rustica in the subgroup of *Ehrlichia chaffeensis*. In nine ticks (five *Am*. *variegatum*, three *Rh*. *microplus* and one *H*. *truncatum*), we detected *Candidatus* Ehrlichia urmitei that was previously observed by our team in *Rh*. *bursa* ticks collected in the Bacque area of France (M. Dahmani, personal communication) (Fig 3). *Candidatus* Ehrlichia urmitei forms an independent and well-supported clade situated between the *E*. *ruminantium* clade and that of *Ehrlichia muris* (Fig 3).

Finally, 15 co-infections (15/378; 4%) were detected by qPCR. All 15 co-infections involved the presence of *R*. *africae*. In *Am*. *variegatum*, ten co-infections (10/15; 66%) were observed with *R*. *africae* plus another pathogen such as *Coxiella burnetii* (1/170; 0.6%), *A*. *centrale* (1/170; 0.6%), *A*. *marginale* (1/170; 0.6%), *Candidatus* Borrelia Africana, *Candidatus* Borrelia ivorensis (3/170; 2%), *Candidatus* Anaplasma ivorensis (2/170; 1%), or *Candidatus* Ehrlichia urmitei (2/170; 1%) as well as *H*. *marginatum rufipes* with *R*. *africae* plus *R*. *aeschlimannii* (2/16; 12%) and in *Rh*. *senegalensis* with *R*. *africae* plus *R*. *aeschlimannii* (1/3; 33%) (Table 3). The access numbers of the sequences of all the potential new species deposited in GenBank are summarized in Table 4.

**Table 4:** New sequences amplified in this study and deposited in GenBank.

Sequences type	Gene	Ascension number
*Candidatus* Borrelia africana TCI22	*flaB*	KT364343
*Candidatus* Borrelia ivorensis TCI140	*flaB*	KT364344
*Candidatus* Borrelia ivorensis TCI351	*flaB*	KT364346
*Borrelia* sp. genotype TCI301	*flaB*	KT364345
*Candidatus* Borrelia africana TCI22	16S rRNA	KT364339
*Candidatus* Borrelia ivorensis TCI140	16S rRNA	KT364340
*Candidatus* Borrelia ivorensis TCI351	16S rRNA	KT364341
*Borrelia* sp. TCI301	16S rRNA	KT364342
*Candidatus* Anaplasma ivorensis TCI50	23S rRNA	KT364326
*Candidatus* Anaplasma ivorensis TCI94	23S rRNA	KT364327
*Candidatus* Anaplasma ivorensis TCI149	23S rRNA	KT364328
*Candidatus* Wolbachia ivorensis TCI113	23S rRNA	KT364329
*Candidatus* Ehrlichia rustica TCI141	23S rRNA	KT364330
*Candidatus* Ehrlichia rustica TCI145	23S rRNA	KT364331
*Candidatus* Ehrlichia rustica TCI167	23S rRNA	KT364332
*Candidatus* Ehrlichia rustica TCI238	23S rRNA	KT364333
*Candidatus* Ehrlichia urmitei TCI148	23Sr RNA	KT364334
*Candidatus* Ehrlichia urmitei TCI230	23S rRNA	KT364335
*Candidatus* Ehrlichia urmitei TCI106	23S rRNA	KT364336
*Candidatus* Ehrlichia urmitei TCI127	23S rRNA	KT364337
*Candidatus* Ehrlichia urmitei TCI166	23S rRNA	KT364338

## Discussion

Domestic animal resources supply some 30% of total human food and agricultural production requirements. They are particularly vital to subsistence and economic development in developing countries as they continually provide essential food products, draught power and manure for crop production and generate income as well as employment for most of the rural poor [63]. However, livestock-associated ticks are often reservoirs or vectors of human vector-borne diseases [18]. Intensification of livestock farming is one cause of the abundance of various vectors and tick-borne diseases. In recent years, the spectrum of tick-borne diseases infecting animals has increased; many of these diseases, such as rickettsioses, borrelioses, Q fever, anaplasmoses, and ehrlichioses, are gaining increasing attention from clinicians and veterinarian [4]. Advances in the development of molecular biology tools facilitate the detection of new bacteria [4,64].

Rickettsioses have been identified in humans, animals and ticks which are considered to be the main vectors of such pathogens as *R*. *africae*, *R*. *aeschlimannii*, and *R*. *massiliae* in sub-Saharan Africa [9]. In our study, rickettsial DNA was found in 49% of ticks collected from cattle. For the first time, the presence of *R*. *aeschlimannii* in ticks in Côte d’Ivoire is shown. This study provides evidence of *R*. *aeschlimannii* infection in 23% of *Hyalomma* ticks including *H*. *marginatum rufipes* (44%) and *H*. *truncatum* (11%). *R*. *aeschlimannii* has not been observed in other tick species. These data support the theory that the *Hyalomma* genus is a main vector and reservoir of *R*. *aeschlimannii*. It was previously reported in 45% to 51% of *H*. *marginatum rufipes* and 6% to 7% in *H*. *truncatum* collected from cows, donkeys, sheep, goats and horses in Senegal [12]. These data are comparable to those of our study. The high prevalence of *R*. *africae* (90%) in *Am*. *variegatum* can be explained by the high transovarial and trans-stadial transmission rates (100%) and a filial infection rate (93%) that was previously demonstrated in *Am*. *variegatum* [20]. This result shows that this tick species acts as a vector but also as a reservoir for *R*. *africae* in Côte d’Ivoire. *R*. *africae* was recently detected in other tick genera including *Rhipicephalus* and *Hyalomma* [12,18,19,57]. In our study, the prevalence of *R*. *africae* is 10% in *Rh*. *microplus* and 9% in *Hyalomma* spp., which is lower than in co-fed *Am*. *variegatum*, suggesting that these ticks are probably not the competent vectors for *R*. *africae*. This bacterium likely infects *Rh*. *microplus* and *Hyalomma* spp. during co-feeding. The first report of the presence of *R*. *massiliae* in Côte d’Ivoire was in *Rhipicephalus* spp. [28]; this is comparable to the detection of *R*. *massiliae* in a *Rh*. *senegalensis* tick found in our study.

*C*. *burnetii* infections have been also reported as being between 0.7% and 6.8% in ticks from cattle in western African countries [51] but not in Côte d’Ivoire where the seroprevalence of *C*. *burnetii* was estimated to be 3% [52]. Here, we show for the first time the presence of *C*. *burnetii* in Côte d’Ivoire, although only in one tick. Most *Borrelia* species such as *B*. *hispanica*, *B*. *duttonii*, and *B*. *crocidurae* detected in Africa, are related to soft ticks. Their main vectors are *Ornithodoros* spp. [65]. To date, *Borrelia* sp. was identified only once in an African hard tick, *Am*. *cohaerens*, in Ethiopia [39]. It has been also reported that *Rhipicephalus* spp. transmits *Borrelia theileri* to cattle, causing bovine borreliosis [18]. In Côte d’Ivoire, we show that *Am*. *variegatum* were infected by three potential new *Borrelia* and *Rh*. *microplus* by one potential new *Borrelia*. The sequences of *Borrelia* sp. (genotype TCI301) were identical to 99% of those of *Borrelia* sp. found in engorged *Rhipicephalus* sp. ticks collected from horse in Brazil (EF141022). Phylogenetic analysis showed that *Borrelia* sp. TCI301 is classified in the relapsing fever group, close to *B*. *crocidurae* and *B*. *hispanica*, two etiological agents of relapsing fever in Africa [26,31]. Blast analysis of the *flaB* gene showed that three new *Candidatus* Borrelia ivorensis and *Candidatus* Borrelia africana borreliae were significantly different to all other borreliae, except for this *Borrelia* sp in *Am*. *cohaerens* in Ethiopia [39]. These potential new *Borrelia* form a new clade between the clades of Lyme disease borreliae and relapsing fever borreliae. Thus, this is the first time that *Borrelia* species have been detected in Côte d’Ivoire and the first time their presence has been confirmed in hard ticks in Africa.

Bacteria from the *Anaplasmataceae* family were previously known to be pathogens of veterinary importance. However, in the three last decades, many human pathogens have been identified in this family [66]. Recently, based on the *rrl* gene, our team developed new tools to identify most bacteria belonging to *Anaplasmataceae* family [47]. These tools combine a qPCR followed by a standard PCR then sequencing, and have been used successfully to amplify DNA from bacteria belonging to *Anaplasma* spp., *Ehrlichia* spp., *Neorickettsia* spp., and *Wolbachia* spp. available in our laboratory [67]. We have successfully amplified *Anaplasmataceae* DNA in 6% of our ticks. For the first time, we have demonstrated the presence of *A*. *marginale*, *A*. *centrale*, *E*. *ruminantium*, and potential novel *Ehrlichia*, *Anaplasma*, and *Wolbachia* spp. in ticks in Côte d’Ivoire. *A*. *marginale* was observed in *Am*. *variegatum* and *Rh*. *microplus*. To the best of our knowledge, *A*. *marginale* has never been reported in Africa in *Rh*. *microplus*. The first report of its presence in Côte d’Ivoire was in 2007, but the exact route of its introduction into this region has not yet been determined [68]. A recent study indicated that the majority of the *Rhipicephalus* (ex-*Boophilus*) spp. collected and identified from farms around Azaguié (Côte d’Ivoire) are *Rh*. *microplus* (96%) [69]. *A*. *centrale* is a species closely related to *A*. *marginale*; this naturally attenuated strain has been used as a live vaccine to prevent severe diseases due to *A*. *marginale senso stricto* strains for 100 years [70]. We identified this species in *Am*. *variegatum*. To the best of our knowledge, *A*. *centrale* has never previously been detected in these ticks. The potential of *Am*. *variegatum* to transmit *A*. *centrale* needs further investigation. *E*. *ruminantium* was previously described in *Am*. *variegatum* which is invasive to cattle attaches to the hooves and cattle remain standing, particularly in the rainy season [71,72]. Recent phylogenetic analyses of *Am*. *variegatum* from Kenya, Mali, Burkina Faso, Ethiopia and the Caribbean show low genetic diversity within this population, suggesting an westward expansion of these ticks and supporting east-west genetic separation, with Caribbean genetic sequences being associated with and often identical to West African haplotypes. The data suggest that *Am*. *variegatum* reached West Africa from Zambia [73]. We have also identified three potential new species, *Candidatus* Anaplasma ivorensis, *Candidatus* Ehrlichia urmitei, and *Candidatus* Ehrlichia rustica. The detection of these potential new species has limitations, as not all previously described species are already molecularly characterized. Indeed, such species as *Anaplasma caudatum*, *Anaplasma bovis*, and *Anaplasma mesaeterum* [74] are incompletely characterized with no strain available and no or few genes sequenced, so the detection of a ‘new’ genotype may, in fact, be the re-discovery of an old, incompletely characterized species. Further studies are required to clarify whether these new genetic variants represent a new species. The other potential new ehrlichiae was closely related to *Ehrlichia* sp. amplified from *Rh*. *bursa* in France. Interestingly, this new species was amplified from two different regions in the world and from different species of ticks (*Rhipicephalus*, *Amblyomma*, and *Hyalomma* spp).

Finally, it is reported that ticks are often co-infected following a blood meal from a co-infected host [75,76]. Recently, mixed infections were reported for the first time in West Africa in feeding ticks and caused mainly by *Rickettsia* spp. and *C*. *burnetii* [18]. In Côte d’Ivoire, for the first time we show multiple co-infections in ticks. These co-infections systematically involved *R*. *africae*. To date, no human cases of anaplasmoses, ehrlichioses, borrelioses, rickettsioses or co-infections have been reported in Côte d’Ivoire. However, these diseases are still little known by clinicians and laboratory diagnostic is lacking in most cases. It is important to continue to study the epidemiological data of such emerging pathogens which may be the source of disease complications in both animals and humans. We provide evidence and demonstrate the endemicity of these different bacteria in the studied regions that have the same characteristics agro-ecological and climatic. Furthermore, these diseases could be a cause of death of unknown origin in rural areas in Côte d'Ivoire [77].
