# Correction: Multiple Pathogens Including Potential New Species in Tick Vectors in Côte d'Ivoire

**DOI:** 10.1371/journal.pntd.0004455

**Published:** 2016-02-05

**Authors:** Cyrille Bilé Ehounoud, Kouassi Patrick Yao, Mustapha Dahmani, Yaba Louise Achi, Nadia Amanzougaghene, Adèle Kacou N’Douba, Jean David N’Guessan, Didier Raoult, Florence Fenollar, Oleg Mediannikov

The following information is missing from the Funding section: This study was supported by UEMOA (P- Z1 – IAD – 002 PAES project number 2100155007376).
